# Administration of a Probiotic Mixture Ameliorates Cisplatin-Induced Mucositis and Pica by Regulating 5-HT in Rats

**DOI:** 10.1155/2021/9321196

**Published:** 2021-09-14

**Authors:** Yuanhang Wu, Jianlin Wu, Zhikun Lin, Qian Wang, Ying Li, Aman Wang, Xiu Shan, Jiwei Liu

**Affiliations:** ^1^Department of Medical Oncology, The First Affiliated Hospital, Dalian Medical University, Dalian, China; ^2^Affiliated Zhongshan Hospital of Dalian University, Dalian, China; ^3^Department of Hepatobiliary Surgery, The First Affiliated Hospital of Dalian Medical University, Dalian, China; ^4^Liaoning CapitalBio Technology Co., Ltd., Dalian, China

## Abstract

Probiotic-based therapies have been shown to be beneficial for chemotherapy-induced mucositis. Previous research has demonstrated that a probiotic mixture (*Bifidobacterium brevis*, *Lactobacillus acidophilus*, *Lactobacillus casei*, and *Streptococcus thermophilus*) can ameliorate chemotherapy-induced mucositis and dysbiosis in rats, but the underlying mechanism has not been completely elucidated. We aimed to determine the inhibitory effects of the probiotic mixture on cisplatin-induced mucositis and pica and the underlying mechanism, focusing on the levels of 5-hydroxytryptamine (5-HT, serotonin) regulated by the gut microbiota. A rat model of mucositis and pica was established by daily intraperitoneal injection of cisplatin (6 mg/kg) for 3 days. In the probiotic+cisplatin group, predaily intragastric injection of the probiotic mixture (1 × 10^9^ CFU/kg BW) was administrated for 1 week before cisplatin injection. This was then followed by further daily probiotic injections for 6 days. Histopathology, pro-/anti-inflammatory cytokines, oxidative status, and 5-HT levels were assessed on days 3 and 6. The structure of the gut microbiota was analyzed by 16S rRNA gene sequencing and quantitative PCR. Additionally, 5-HT levels in enterochromaffin (EC) cells (RIN-14B cell line) treated with cisplatin and/or various probiotic bacteria were also determined. The probiotic mixture significantly attenuated kaolin consumption, inflammation, oxidative stress, and the increase in 5-HT concentrations in rats with cisplatin-induced intestinal mucositis and pica. Cisplatin markedly increased the relative abundances of *Enterobacteriaceae_other*, *Blautia*, *Clostridiaceae_other*, and members of *Clostridium* clusters IV and XIVa. These levels were significantly restored by the probiotic mixture. Importantly, most of the genera increased by cisplatin were significantly positively correlated with colonic 5-HT. Furthermore, in vitro, the probiotic mixture had direct inhibitory effects on the 5-HT secretion by EC cells. The probiotic mixture protects against cisplatin-induced intestine injury, exhibiting both anti-inflammatory and antiemetic properties. These results were closely related to the reestablishment of intestinal microbiota ecology and normalization of the dysbiosis-driven 5-HT overproduction.

## 1. Introduction

Cisplatin is a chemotherapy agent used for the first-line treatment of the majority of cancer patients [1]. Although clinical trials have shown effectiveness, unfavorable cytotoxic side effects are a huge hurdle that impedes the clinical application of otherwise beneficial cisplatin-based treatments [2–4]. Intestinal mucositis, a serious chemotherapy-induced side effect, is characterized by local accumulation of inflammatory cells [5], cell loss in the epithelial barrier [6], increased oxidative stress [7], and reduced gastrointestinal digestive enzyme activities [8]. Clinical manifestations include nausea, vomiting, ulceration, bloating, anorexia, constipation, severe diarrhea, and subsequent weight loss [9–11], causing potentially life-threatening complications. Improved management of cisplatin-induced mucositis and nausea/vomiting may help avoid reductions in and discontinuation of chemotherapy among patients.

Advances during the past three decades have elucidated some of the mechanisms by which chemotherapeutic agents induce side effects. Among these mechanisms, the effect of the neurotransmitter 5-hydroxytryptamine (5-HT, serotonin) plays an important part in chemotherapy-induced mucositis [9]. Cisplatin treatment may cause free radical generation, leading to localized exocytotic release of 5-HT from the enterochromaffin (EC) cells [10]. Thereafter, 5-HT interacts with 5-HT3 receptors on vagal afferent terminals in the wall of the bowel and transmits the stimulus to the brain, resulting in emesis [10]. 5-HT also regulates interstitial cells of Cajal (ICCs) in the gastrointestinal (GI) tract [12] and aquaporin 3 (AQP3) expression in the colon [13], which is closely related to constipation and diarrhea. In addition, increased intestinal 5-HT level coincides with deregulation of the mucosal immune system, which in turn exacerbates damage [14]. Currently, most drugs used as prophylaxis for chemotherapy-induced side effects belong to the classes of 5-HT3 receptor antagonists [15]. As a result, the quality of life for cancer patients has improved to a certain degree. However, these antagonists can also cause central nervous system (CNS) side effects, such as headaches, dizziness, and abnormal vision [16]. Therefore, adjuvant therapy targeting the biosynthesis of 5-HT by EC cells is a promising way to ameliorate chemotherapy-induced mucositis.

More than 90% of the total 5-HT in the human body is synthesized, stored, and released by EC cells in the intestinal mucosa [17]. 5-HT biosynthesis by EC cells was recently found to be modulated by human gut bacteria, such as *Clostridium* spp. [18]. This suggests a critical interaction between the gut microbiota and chemotherapy-induced GI complications. Disturbance of the commensal microbial structure, so-called dysbiosis, will inevitably influence EC cells and eventually affect the 5-HT-mediated stimulation of the GI tract and brain [19]. In recent years, several studies have explored gut dysbiosis in patients taking cisplatin-based chemotherapy [8, 20–22]. However, few studies have systematically evaluated the possible correlation between gut dysbiosis and 5-HT-mediated GI mucositis. In addition, whether reduction of chemotherapy-induced dysbiosis by probiotics can ameliorate the 5-HT overproduction by EC cells remains unclear.

Although rats do not vomit [23], they exhibit pica behavior (eating of nonnutritive substances such as kaolin) in response to a variety of emetogenic stimuli, which can be used as a proxy variable for nausea and vomiting in rat models [24, 25]. In this study, we administered a probiotic mixture (*Bifidobacterium brevis* DM8310, *Lactobacillus acidophilus* DM8302, *Lactobacillus casei* DM8121, and *Streptococcus thermophilus* DM8309) to rats with cisplatin-induced mucosal damage and pica. We aimed to investigate its effects on inflammation, oxidative stress, gut microbiota modification, and the levels of serum and colonic 5-HT in the rats. Our results showed that the probiotic mixture ameliorated cisplatin-induced mucositis and pica in rats and normalized the dysbiosis-driven 5-HT overproduction. These effects may be closely related to the effects of the probiotic mixture on gut dysbiosis in the cisplatin-treated rats.

## 2. Materials and Methods

### 2.1. Bacterial Strains and Drugs

A probiotic mixture, including the following strains: *Bifidobacterium brevis* DM8310, *Lactobacillus acidophilus* DM8302, *Lactobacillus casei* DM8121, and *Streptococcus thermophilus* DM8309, was provided by Dalian Medical University and prepared as described in a previous study [26]. Briefly, the strains were cultured separately on solid media under anaerobic condition, at 37°C for 24 h. After that, single colonies were picked and inoculated into MRS liquid medium and cultured for 24 hours. The bacteria were then mixed by centrifugation at 5,000 × *g*, 4°C for 2 min, and resuspended in 2 mL PBS before administration to rats. Cisplatin was obtained from Qilu Pharmaceutical Co., Ltd. (China) and dissolved in saline. Kaolin, acquired from China Pharmaceutical Chemical Reagents Co., Ltd. (China), was prepared and processed as previously described [21].

### 2.2. Animal Study

Sprague-Dawley (SD) male rats aged 8 weeks old and weighing 180~200 g each were supplied by the Specific Pathogen Free animal centre of Dalian Medical University and were kept on a 12 h light/dark cycle with access to food and water ad libitum in a temperature-controlled room (25°C). The kaolin pellets were introduced into the rats 6 days prior to cisplatin injection. Most of the rats stopped taking Kaolin on the third day, and those that were still interested in kaolin were excluded. The remaining rats were randomly divided into 3 experimental groups (*n* = 10 each), and toxicity was induced by daily intraperitoneal injection of cisplatin to rats with the dosage of 6 mg/kg for 3 days. The rats in the control group (Con) were subjected to saline (0.9% NaCl) injection every day and saline daily by gavage. The cisplatin group (Cis-p) received saline daily by gavage. The probiotic+cisplatin group (PM) was given cisplatin injection daily for 3 days and administration of probiotic mixture (1 × 10^9^ CFU/kg-BW) daily for 6 days. Probiotic mixture was administered one week before cisplatin injection. The general conditions of rats were examined every day, including activities, fur, appetite, breath, and stool. The body weight and consumption of kaolin were recorded every 24 h until the rats were sacrificed after anesthesia with sodium pentobarbital on the assigned day of each experiment. The small intestine (corresponding to the jejunum and ileum, resp.) and colon were removed. All animal work was performed according to the laboratory's animal ethics guidelines, and protocols were approved by the Dalian Medical University Institutional Animal Care and Use Committee (SYXK [Liao] 2017-0003).

### 2.3. Intestinal Histology and Immunofluorescence Staining

The colon of the rat was cut into distal, medial, and proximal sections, and 1 cm of different sections was fixed in 4% paraformaldehyde overnight at 4°C and then embedded in paraffin, and 4 *μ*m thick sections were prepared. For histological analysis, sections were stained with hematoxylin/eosin (HE) and analyzed. The severity of colon damage was semiquantitatively scored according to a histological scoring scale previously described [27]. For double-label immunofluorescence immunohistochemistry, slides were blocked with 10% NDS containing 0.3% Triton X-100 at 4°C for 2 h and incubated with rabbit anti-mouse CgA (1 : 500, Abcam) in combination with a rat anti-mouse 5-HT (1 : 50; Abcam) antibody at 20°C. After washing, the sections were incubated with a mixture of Alexa Fluor 488-conjugated donkey anti-rabbit IgG and Alexa Fluor 594-conjugated donkey anti-rat IgG (Invitrogen, Rockford, IL, USA; 1 : 1,000) secondary antibodies at 20°C for 3 h while protected from light. The sections were then mounted on glass slides and finally embedded with Fluoromount/Plus (K048, Diagonostic Biosystems, Pleasanton, CA, USA) after drying at room temperature for 30 min.

### 2.4. ELISA

The levels of lipopolysaccharides (LPS, USCN, USA), malondialdehyde (MDA, Nanjing Jiancheng Bioengineering Institute, China), serotonin, and cytokines (USCN, USA) including TNF-*α*, IL-6, and IL-10 in sera and/or supernatant of tissue homogenates were detected by ELISA according to the manufacturer's instructions. Readings from tissue samples were normalized to total protein content as detected by the BCA assay (Thermo Pierce). Data that compiled across multiple experiments are expressed as concentrations normalized to controls within each experiment.

### 2.5. The Quantitative Real-Time PCR (qPCR) Detection

The extraction of total RNA and the synthesization of the complementary DNA (cDNA) were performed as previously described [28]. For detection of rat genes, amplification was performed in triplicates in 384 well plates (QuantStudio 6 Flex Real-Time PCR System) using ChamQ Universal SYBR qPCR Master Mix (Vazyme). The expression levels were calculated using the comparative 2^−△△ct^ method. For detection of fecal bacterial groups of rats, amplification and detection were performed as previously described [26]. Bacterial quantity was expressed as log10 bacteria/g of fecal content. qPCR primers are listed in Table 1.

### 2.6. 16S rRNA Gene Sequencing and Analysis

The fecal metagenomic DNA of rats was extracted using the QIAamp DNA Stool Mini Kit (Qiagen, Hilden, Germany). The primer pair 520F/802R was used to amplify the V3-V4 hypervariable region of the bacterial 16S rDNA from bacteria in rat feces of six groups (*n* = 4-5 each). HiSeq sequencing and data analysis were subsequently performed using a method described previously [29]. Operational taxonomic units (OTUs) present in 50% or more of the fecal samples were identified as core OTUs. Principal component analysis (PCA) was then conducted according to the distance matrices to analyze the diversity between groups.

### 2.7. RIN-14B In Vitro Culture Experiments

RIN-14B cells were purchased from ATCC (ATCC No. CCL 89) and seeded into 24-well plates at a density of 2 × 10^5^ cells/0.5 mL RPMI1640 medium (Invitrogen-Japan) with 10% FCS (Thermo Fischer Scientific), 1% penicillin and streptomycin (Thermo Fisher Scientific) at 37°C, and 5% CO_2_. 1 mL of the bacteria culture broths (OD600 = 1.5) was centrifuged at 2500 rpm for 5 minutes, and supernatants were filtered through 0.2 *μ*m pore syringe filters. Cultured RIN-14B cells were incubated with different bacterial filtrates for 1 h at 37°C. For cisplatin treatment, cells were incubated with cisplatin (1 *μ*g/mL), passed through 0.2 *μ*m pore syringe filters, while for the cisplatin combined with PM treatment, cells were incubated with cisplatin (1 *μ*g/mL) and 1 mL probiotic mixture. After incubation, the supernatant was collected, which was then centrifuged 6000 × *g* for 5 min and frozen for downstream 5-HT analysis.

### 2.8. Statistical Analysis

All quantitative data were expressed as the mean ± standard error of the mean (SEM). Statistical analyses were performed using two-tailed, unpaired Student's *t*-test for comparisons of two groups when data obey normal distribution and even variance and using one-way ANOVA followed by the Kruskal-Wallis rank sum test for multigroup comparisons. Pearson's correlation test was used to analyze the correlation between the differential microbes and colon 5-HT level. Significance was set at ^∗^*p* < 0.05, ^∗∗^*p* < 0.01, ^∗∗∗^*p* < 0.001, and ^∗∗∗∗^*p* < 0.0001.

## 3. Results

### 3.1. Probiotic Mixture Ameliorates Cisplatin-Induced Pica and Mucositis in Rats

**Figure 1:**
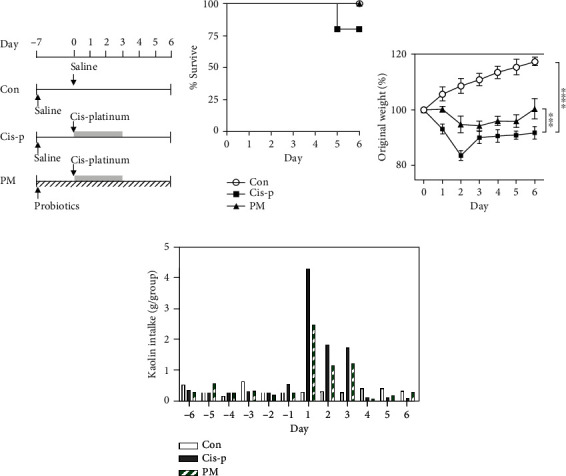
Effects of probiotic mixture on cisplatin-induced mucositis and pica in rats (*n* = 5): (a) experimental setup, (b) survival rate, (c) body weight, and (d) kaolin consumption. Con: normal control group; Cis-p: cisplatin-treated model group; PM: probiotic mixture- and cisplatin-treated group.

We established a rat model of pica and intestinal mucositis by intraperitoneally injecting cisplatin. The experiment design is shown in Figure 1(a). During the 6 d experiment, one rat died after cisplatin treatment in the Cis-p group on day 5 (Figure 1(b)). No other rats died. The body weight and kaolin consumption level (indicating the degree of pica) were measured. The body weight of rats in the Cis-p group decreased (*p* < 0.0001 vs. the Con group, Figure 1(c)). The probiotic mixture ameliorated the decreased body weight to a certain extent (*p* = 0.0002 vs. the Cis-p group, Figure 1(c)). Before the first injection of cisplatin, the rats in the different experimental groups consumed a similar amount of kaolin (*p* > 0.05 between each pair of groups). After injection of cisplatin, the kaolin consumption was significantly increased in the cisplatin-only rats compared to the control rats. Kaolin consumption in the PM group decreased to a certain extent, indicating that the probiotic mixture can ameliorate cisplatin-induced pica in rats (Figure 1(d)).

### 3.2. Probiotic Mixture Improves the Intestinal Barrier Function in Cisplatin-Induced Mucositis

**Figure 2:**
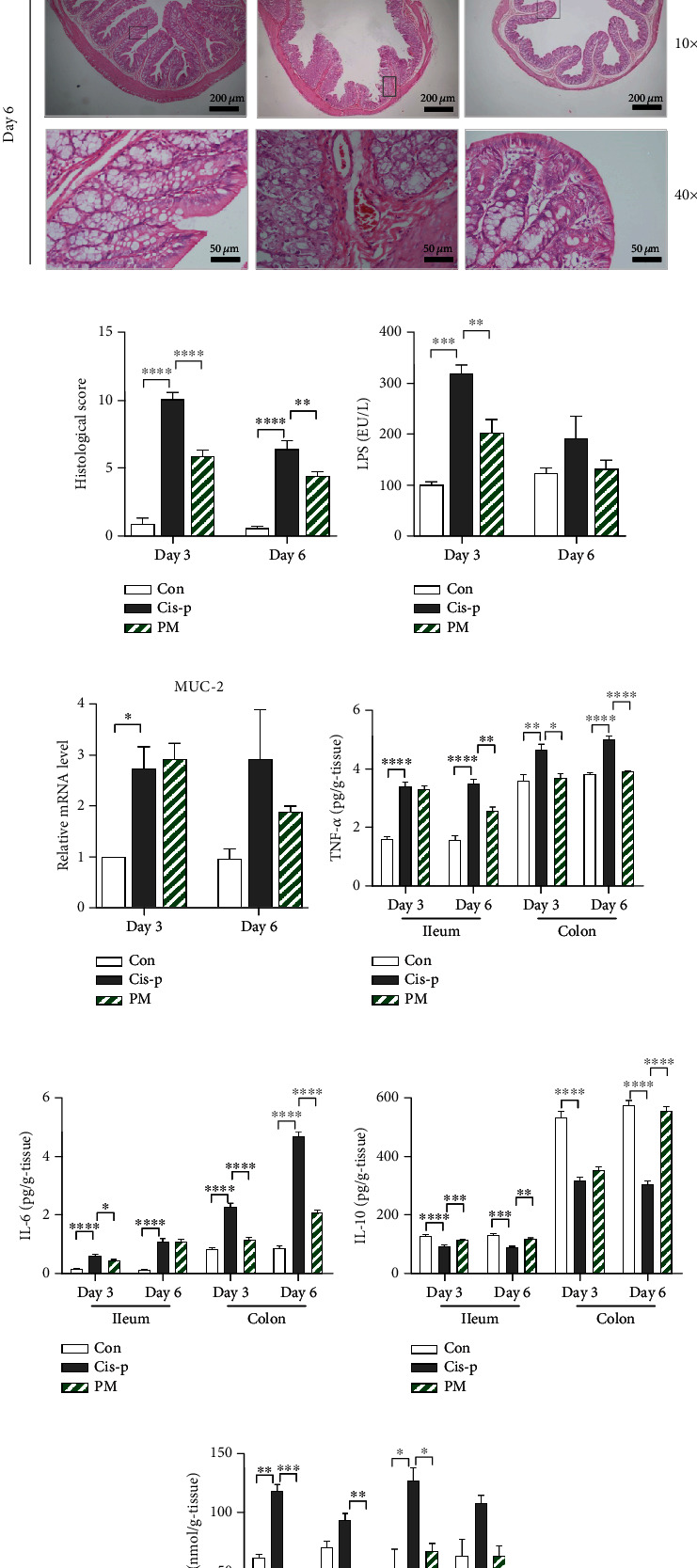
Effects of probiotic mixture on the intestinal barrier function in cisplatin-induced mucositis. (a) H&E staining of rat colon sections. (b) Histopathological analysis of H&E-stained sections. (c) Serum LPS were detected by ELISA. (d) Relative mRNA expression of mucin-2 (MUC-2) in colon tissues of rats, detected by quantitative PCR. Levels of tumor necrosis factor- (TNF-) *α* (e), interleukin- (IL-) 6 (f), IL-10 (g), and malondialdehyde (MDA) (h) in the ileum and colon. Data are expressed as mean ± SEM (*n* = 3-5): ^∗^*p* < 0.05, ^∗∗^*p* < 0.01, ^∗∗∗^*p* < 0.001, and ^∗∗∗∗^*p* < 0.0001.

Morphologic observation of the colon tissue of rats after cisplatin treatment revealed severe mucosal lesions with villous atrophy, irregular arrangement and architecture of the epithelial cell, and crypt disruption in the colon mucosa compared to the control rats. Compared to the cisplatin-only rats, the probiotic mixture protected the colon mucosa from cisplatin-induced injury by improving these factors (Figure 2(a)). The histological score of the colon in rats treated with cisplatin was higher compared with control and the probiotic mixture administrated rats (day 3: Con vs. Cis-p, *p* < 0.0001; Cis-p vs. PM, *p* < 0.0001; day 6: Con vs. Cis-p, *p* < 0.0001; Cis-p vs PM, *p* = 0.0082; Figure 2(b)). A significantly elevated serum lipopolysaccharide (LPS) level, which indicates gut permeability, was found in the cisplatin-only rats (*p* = 0.0003), and the probiotic mixture significantly inhibited this increase (*p* = 0.0088; Figure 2(c)). Mucin-2 (MUC-2) is the key constituent of the mucosal barrier that protects the mucosal epithelial layer. The mRNA expression levels of MUC-2 in the colon were significantly increased in the Cis-p group on day 3 (*p* = 0.0142; Figure 2(d)) and nonsignificantly decreased in the PM group on day 6. Additionally, compared to the Cis-p group, the probiotic mixture led to further beneficial effects on both days 3 and 6, such as reducing the elevated intestinal level of inflammatory tumor necrosis factor- (TNF-) *α* (ileum: *p* = 0.0025 [day 6]; colon: *p* = 0.0107 [day3]; *p* < 0.0001 [day 6]; Figure 2(e)) and interleukin- (IL-) 6 (ileum: *p* = 0.0136 [day 3]; colon: *p* < 0.0001 [day3]; *p* < 0.0001 [day 6]; Figure 2(f)) and promoting the secretion of IL-10 (ileum: *p* = 0.0008 [day 3]; *p* = 0.006 [day 6]; colon: *p* < 0.0001 [day 6]; Figure 2(g)). These results suggested that the probiotic mixture has an anti-inflammatory effect on cisplatin-induced intestinal mucositis in rats. Malondialdehyde (MDA), a product of lipid peroxidation, is an oxidative stress biomarker. Cisplatin increased the MDA level in intestinal tissues on both days 3 and 6, while the probiotic mixture significantly decreased the MDA concentration (ileum: *p* = 0.0008 [day 3]; *p* = 0.006 [day 6]; colon: *p* = 0.0001 [day 3]; Figure 2(h)). Together, these data indicated that the probiotic mixture had significant effects on the intestinal barrier functions, including the mechanical, immune, and chemical barrier functions.

### 3.3. Probiotic Mixture Reduces Serum and Colonic 5-HT Overproduction

**Figure 3:**
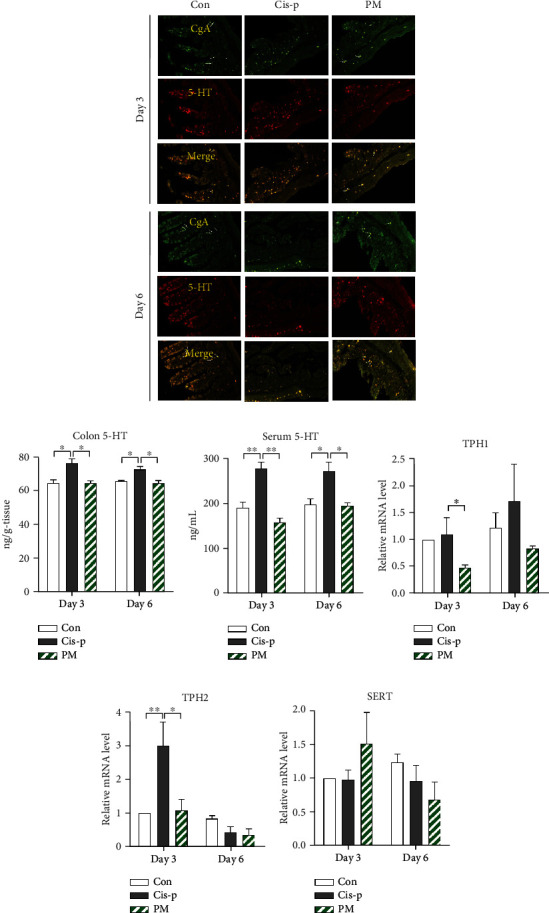
Effects of probiotic mixture on cisplatin-induced colonic 5-HT biosynthesis in rats. (a) Representative images of colons with chromogranin A (gastrointestinal endocrine cell marker, green) and 5-HT (red) staining. Levels of colonic (b) and serum (c) 5-HT. Colonic expression of TPH1 (d), TPH2 (e), and SERT (f) relative to *β*-actin. Data are normalized to expression levels in the control rats on day 3. Data are expressed as mean ± SEM (*n* = 4-5): ^∗^*p* < 0.05 and ^∗∗^*p* < 0.01.

As cisplatin-induced emesis is associated with an increase in 5-HT concentration, produced by EC cells, we conducted immunofluorescent staining of rat colon tissues in the three groups and also determined the colonic and serum levels of 5-HT. Double staining of chromogranin A (GI endocrine cell marker) and 5-HT showed that cisplatin-induced increases in 5-HT were localized to the colonic EC cells (Figure 3(a)). The levels of 5-HT in the colon and serum of the cisplatin-only rats were significantly increased in comparison to the control group (colon: *p* = 0.0171 [day 3]; *p* = 0.0327 [day 6]; serum: *p* = 0.0056 [day 3]; *p* = 0.0277 [day 6]; Figures 3(b) and 3(c)), and the probiotic mixture reduced the cisplatin-induced increases in colonic and serum levels of 5-HT, with significant differences on both days 3 and 6 (colon: *p* = 0.018 [day 3]; *p* = 0.0148 [day 6]; serum: P =0.0012 [day 3]; *P* = 0.0238 [day 6]; Figures 3(b) and 3(c)).

To investigate whether the inhibitory effects of the probiotic mixture on 5-HT levels in rats are due to the regulation of 5-HT synthesis or transport, we assessed the mRNA expression of tryptophan hydroxylases (TPH1 and TPH2) and the serotonin reuptake transporter (SERT) by quantitative real-time PCR. After cisplatin administration, TPH2 expression increased significantly in the colon of rats compared to the control group on day 3 (*p* = 0.008, Figure 3(e)). The probiotic mixture greatly downregulated TPH1 (*p* = 0.042) and TPH2 (*p* = 0.013) in the colon of rats compared to the levels in the cisplatin-only rats (Figures 3(d) and 3(e)). The expression level of SERT in the colon of rats showed no significant differences (Figure 3(f)).

### 3.4. Regulation of Cisplatin-Induced Gut Dysbiosis in Rats by Probiotic Mixture Is Associated with Changes in the 5-HT Level

**Figure 4:**
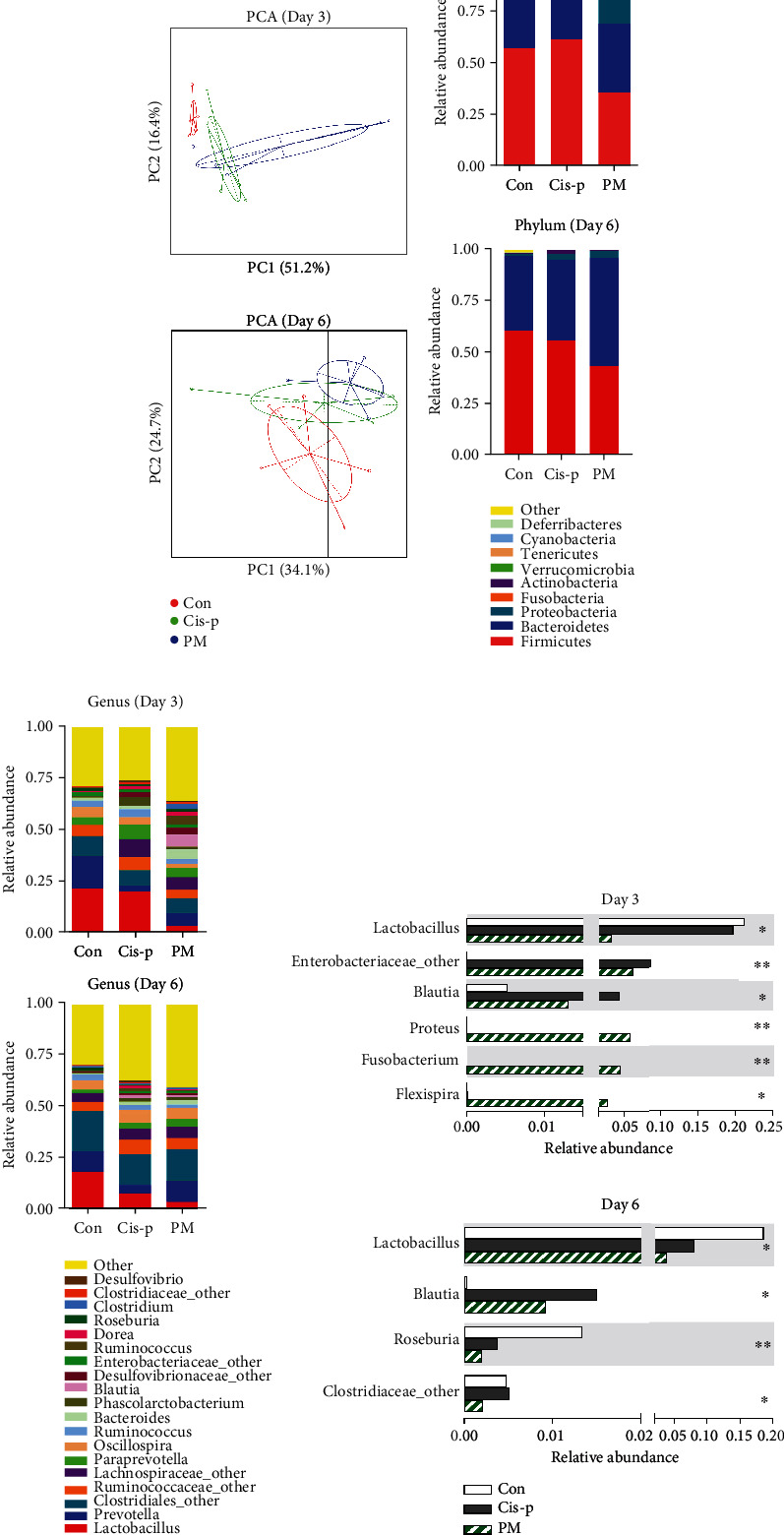
Probiotic mixture modulated intestinal bacterial composition in cisplatin-treated rats (*n* = 4-5). (a) Principal component analysis (PCA) among different samples. (b) Mean abundances in the gut microbiota at the phylum level. (c) Bar charts of the gut microbiota at the genus level. (d) Species differences among the three groups at the genus level. ^∗^*p* < 0.05 and ^∗∗^*p* < 0.01.

To reveal the effects of the probiotic mixture on the gut microbiota during cisplatin-induced mucositis, sequencing targeting the V3–V4 region of the 16S rDNA was performed. Principal component analysis (PCA) revealed that the microbiota in the three groups were distinct from each other on day 3, especially regarding principal component 1 (PC1; 51.2%) (Figure 4(a)), indicating that cisplatin causes variation in the gut flora.

To further compare the gut microbiota composition among the three groups, histograms of the relative abundances were constructed, as shown in Figure 4(b) for phyla and Figure 4(c) for genera. The two most abundant bacterial phyla in rat were *Firmicutes* and *Bacteroidetes*. In the cisplatin-only rats, the relative abundance of *Firmicutes* increased (Figure S1A), while the relative abundance of *Bacteroidetes* decreased (Figure S1B), and the ratio of *Firmicutes* to *Bacteroidetes* (*F*/*B*) increased (Figure S1C), compared to the other two groups on day 3. Significant differences at the genus level were also observed (Figure 4(d)). Notably, the cisplatin-only rats had greater relative abundances of *Enterobacteriaceae_other* and *Blautia* on day 3 and *Blautia* and *Clostridiaceae_other* on day 6 but lower abundances of *Lactobacillus* (day 3) and *Roseburia* (day 6) than in the control group, suggesting that cisplatin closely affects the microbiota composition. Interestingly, in the PM group, *Proteus*, *Fusobacterium*, and *Flexispira* were clearly increased on day 3 compared to those in the cisplatin-only group. The relative abundances of *Lactobacillus*, *Enterobacteriaceae_other*, *Blautia*, *Roseburia*, and *Clostridiaceae_other* decreased.

### 3.5. Probiotic Mixture Reduces Specific Bacteria That Promote Colonic 5-HT Biosynthesis In Vivo and Inhibit 5-HT Secretion by EC (RIN-14B) Cells In Vitro

**Figure 5:**
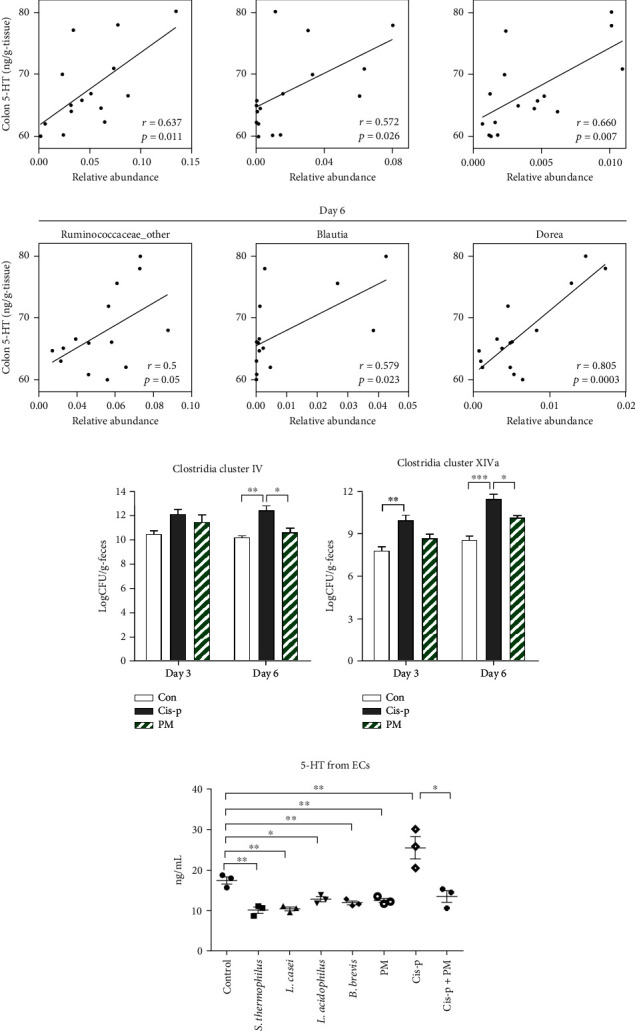
Probiotic mixture reduced 5-HT-associated bacteria and inhibited 5-HT secretion by RIN-14B cells. (a) Correlations between the abundances of differential species and colonic 5-HT level. Abundances of Clostridium cluster IV (b) and XIVa (c) in the feces of rats in different groups detected by quantitative PCR. Data are presented as the mean logarithm of bacterial colony-forming units (CFU) per gram of feces. (d) Levels of 5-HT released from RIN-14B cells after exposure to various bacteria or cisplatin. Data are expressed as mean ± SEM (*n* = 3–5): ^∗^*p* < 0.05, ^∗∗^*p* < 0.01, and ^∗∗∗^*p* < 0.001.

According to our finding that 5-HT levels were decreased in the colons of probiotic+cisplatin rats compared to the cisplatin-only rats, we therefore hypothesized that specific microbes are associated with affecting the host 5-HT pathways. Using Pearson's correlation analysis, bacteria that were significantly related to the colonic 5-HT variation were identified and plotted. We found that the genera *Ruminococcaceae_other*, *Blautia*, *Ruminococcus*, and *Dorea* were all significantly positively correlated with colonic 5-HT (Figure 5(a)). Relative quantification of selected 5-HT-associated bacterial groups was performed by quantitative real-time PCR. The results further revealed that the members of *Clostridium* clusters IV and XIVa (butyrate producers) were significantly increased in the cisplatin-only rats compared to the control rats. The PM group showed decreased abundances of *Clostridium* cluster IV and XIVa compared to the cisplatin-only group, especially on day 6 (*p* = 0.015, Figure 5(b); *p* = 0.03, Figure 5(c)).

To determine whether the protective effects of the probiotic mixture were directly associated with the inhibition of 5-HT secretion by EC cells, we further subjected RIN-14B cells to no treatment (control), one of the four bacteria in the probiotic mixture or the mixture itself, cisplatin only, or cisplatin plus the probiotic mixture. We found that all four bacteria and the mixture itself significantly decreased 5-HT secretion compared to the control cells (*S. thermophilus*, *p* = 0.003; *L. casei*, *p* = 0.002; *L. acidophilus*, *p* = 0.013; *B. brevis*, *p* = 0.005; PM, *p* = 0.009; Figure 5(d)). Furthermore, the probiotic mixture significantly reduced cisplatin-induced 5-HT overproduction (Cis-p vs. control, *p* = 0.002; PM vs. Cis-p, *p* = 0.018; Figure 5(d)). This suggested that the probiotic mixture not only reduced 5-HT-associated bacteria but also had direct inhibitory effects on 5-HT biosynthesis in EC cells.

## 4. Discussion

In this study, the inhibitory effects of a probiotic mixture on cisplatin-induced mucositis and pica in rats were investigated. We found that the underlying mechanisms involved gut microbiota-induced alteration of gut-derived 5-HT biosynthesis.

Chemotherapy-induced mucositis and nausea/vomiting are common and major debilitating side effects of chemotherapy, which severely affects quality of life among cancer patients, even reducing their compliance with chemotherapy [30]. Cisplatin, a cytotoxic agent, is widely prescribed in chemotherapy regimens for various human cancers due to its affinity for DNA [31]. Cisplatin causes both acute and delayed GI tract disorders (<24 and >24 hours after administration, respectively) [32]. Our results are consistent with those of prior studies in which cisplatin caused significant mucositis and pica in rats [8, 33–35]. Another study revealed that treatment with the probiotic mixture used in our study (*Bifidobacterium brevis* DM8310, *Lactobacillus acidophilus* DM8302, *Lactobacillus casei* DM8121, and *Streptococcus thermophilus* DM8309) alleviates 5-fluorouracil-induced mucositis in rats [26]. Our study demonstrated that this probiotic mixture also attenuated cisplatin-induced mucositis and pica in rats, which involved reducing inflammation and oxidative stress. A clinical study found that treatment with *B. breve* ameliorated intestinal mucositis in pediatric cancer patients taking chemotherapy [36]. *L. acidophilus* and *L. casei* both significantly improved the inflammatory and functional aspects of 5-fluorouracil-induced intestinal mucositis in mice [37, 38]. Furthermore, oral ingestion of *S. thermophilus* alleviated the symptoms of methotrexate-induced mucositis [39]. Thus, the probiotic mixture used in this study potentially improves GI function.

Intestinal barrier integrity is critical for intestinal function. Emerging data suggest that chemotherapy exerts detrimental effects on intestinal permeability, contributing to inflammation [40]. We detected histological abnormalities in the colon of mice treated with cisplatin, which were reversed by the probiotic mixture (Figure 2). In addition, compared to the cisplatin-only group, as indicated by the decreased endotoxin LPS in the serum. Although MUC-2 has been shown to have protective capacities, we found that it was upregulated during cisplatin-induced mucositis, especially on day 3, and it reverted to a normal level by day 6 in the probiotic+cisplatin group. MUC-2 upregulation may be a counterreaction by the intestine to protect against mucositis [41], which was also observed in methotrexate-induced damage [42, 43]. The mechanism may involve short-chain fatty acids (SCFAs) regulating prostaglandin production, thus stimulating MUC-2 expression in intestinal epithelial cells [44]. Our results also indicated that cisplatin-induced inflammation was attenuated by the probiotic mixture. The probiotic mixture inhibited cisplatin-induced generation of TNF-*α* and IL-6 and promoted the secretion of IL-10. In the cisplatin-only rats, the increase in inflammatory cytokines may be attributable to the degradation of the epithelial barrier; furthermore, this may trigger dysbiosis and elicit secondary inflammation, ultimately resulting in intestinal mucositis [45]. Oxidative stress is also a critical component of cisplatin-induced intestinal injury [46]. There is evidence that some lactic acid bacteria (*Bifidobacterium*, *Lactobacillus*, *Lactococcus*, and *Streptococcus thermophilus*) exerted antioxidant activity [47]. Our results also revealed that the probiotic mixture (*Bifidobacterium*, *Lactobacillus*, and *Streptococcus thermophilus*) reduced oxidative stress by decreasing MDA concentrations in both colon and ileum tissues. Thus, all the data suggested that the probiotic mixture has potentially anti-inflammatory and antioxidant activities.

It has been suggested that acute GI tract disorders involve 5-HT secretion from EC cells, which are a subset of enteroendocrine cells that reside within the intestinal mucosa [48]. A rate-limiting enzyme in 5-HT biosynthesis is TPH, which is present in two isoforms (TPH1 and TPH2). TPH1 is primarily expressed in the EC cells of the gut, whereas TPH2 is expressed in serotonergic neurons [49]. TPH expression can be considered an indirect biomarker of 5-HT synthesis. 5-HT is inactivated by SERT-mediated uptake into mucosal epithelial cells or enteric neurons [50]. Therefore, TPH and SERT are important for determining the 5-HT concentration and dynamics. A preliminary study revealed that cisplatin significantly increased TPH1 mRNA expression, but not TPH2 and SERT mRNA expression, in ileal tissue [51]. Another study demonstrated that cisplatin increased TPH1 and TPH2 mRNA expression in the ileum and medulla oblongata, respectively, indicating that cisplatin accelerates 5-HT synthesis through upregulating TPH, but has no effect on SERT [34]. We found that cisplatin significantly increased TPH1 and TPH2 levels in the colon but did not affect the SERT level. Thus, the increase in colonic TPH activity after cisplatin administration may be due to an increase in the number of EC cells and enteric neurons that express TPH1 and TPH2 mRNA. Furthermore, the probiotic mixture significantly inhibited the increase in 5-HT levels in the colon and serum, probably because it inhibited 5-HT synthesis in the colon (Figure 3).

The gut microbiota plays an important role in promoting levels of colonic and serum 5-HT. Yano et al. [18] found that germ-free mice exhibited significantly decreased levels of colonic and serum 5-HT compared to specific pathogen-free (SPF) controls, and the study suggested that the microbiota regulated 5-HT metabolism primarily by affecting host colonic EC cells. Gut dysbiosis is known to be involved in the pathogenesis of chemotherapy-induced mucositis, and restoring gut microbiota homeostasis may accelerate intestinal healing [40, 52, 53]. The probiotic mixture reversed cisplatin-induced gut dysbiosis—as indicated by the increased *F*/*B* ratio [54, 55]. We found that cisplatin treatment caused a significant increase in several gut bacterial taxa such as *Enterobacteriaceae_other*, *Blautia*, and *Clostridiaceae_other*, which were significantly restored by the probiotic mixture. Similar trends were observed in the abundances of *Ruminococcaceae_other*, *Ruminococcus*, and *Dorea*, although the differences were not significant. Notably, *Ruminococcaceae_other*, *Blautia*, *Ruminococcus*, and *Dorea* were all significantly positively correlated with colonic 5-HT (Figure 5). The abundances of *Enterobacteriaceae* and *Blautia* have been reported to be increased in chemotherapy-induced dysbiosis [56–58]. Consistent with our results, *Ruminococcaceae* and *Ruminococcus* have been reported to be positively correlated with 5-HT [59, 60]. However, we also found that *Proteus*, *Fusobacterium*, and *Flexispira*, as putative gastrointestinal pathogens [61–63], were clearly increased in the PM group on day 3 compared with the other two groups (Figure 4(d)). We considered that the sample size in the Illumina Hiseq sequencing was not enough; further studies based on a larger number of samples should be conducted to confirm the results. Importantly, the gut microbiota compositions of the three groups are different on days 3 and 6, which due to the gut epithelium have remarkable self-renewal capacity, and the intestinal microbiota also has the recovery ability to repair damaged mucosal barrier [64, 65]. Furthermore, the probiotic mixture accelerated recovery of cisplatin-induced intestinal damage.

It is worth noting that SCFAs are well-known metabolites produced by the microbiota. They have been suggested to influence 5-HT secretion. The SCFAs' butyrate and acetate, which are produced in abundance by distal gut microbes in vivo, significantly affect enteric 5-HT production by promoting TPH1 transcription [66]. *Clostridia* are anaerobic *Firmicutes* that produce a large array of metabolites. Moreover, *Clostridium* clusters IV and XIVa (also known as the *Clostridium leptum* and *Clostridium coccoides* groups, respectively) contain butyrate-producing species [67]. We therefore explored the link between *Clostridium* clusters IV and XIVa and cisplatin-induced intestinal injury. Riezzo et al. reported that a clinically relevant combination of irinotecan and 5-FU decreased *Clostridium* cluster IV and increased *Clostridium* cluster XIVa [68]. However, other studies showed that both an irinotecan regimen and 5-FU decreased the abundances of *Clostridium* cluster XIVa [26, 56]. Due to different drugs and different sampling times, our results demonstrated that cisplatin significantly increased members of *Clostridium* clusters IV and XIVa, which produce butyrate and thereby promote 5-HT secretion by EC cells. Therefore, we speculated that the probiotic mixture ameliorated cisplatin-induced mucositis and pica in rats partly by normalizing the dysbiosis-driven 5-HT overproduction. Additionally, probiotic bacterial species and probiotic supplements have a tendency to directly reduce colonic 5-HT [69, 70]. We confirmed that the probiotic mixture also had direct inhibitory effects against 5-HT biosynthesis in EC cells in vitro. To sum up, the probiotic mixture supplementation in cisplatin-treated rats reestablished the intestinal ecosystem, particularly the populations of probiotic bacteria, which in turn attenuated the increased 5-HT.

## 5. Conclusion

In this study, the protective effects of a probiotic mixture against cisplatin-induced mucositis and pica and the potential underlying mechanisms were evaluated. The probiotic mixture significantly ameliorated kaolin consumption, inflammation, oxidative stress, and the increased 5-HT concentrations caused by cisplatin. Furthermore, the probiotic mixture mitigated the gut dysbiosis and attenuated the altered metabolic profiles induced by cisplatin. The mechanism underlying the attenuation of cisplatin-induced intestinal injury may involve the probiotic mixture modulating the dysbiosis-driven 5-HT overproduction. The findings of this study indicate that the probiotic mixture may be useful for treating intestinal injury induced by cisplatin-based chemotherapy. However, more optimized study design and a larger sample size would benefit future studies. Further studies are required to fully understand the in-deep mechanisms of how the probiotic mixture supplementation ameliorates the cisplatin-induced mucositis and pica.

## Figures and Tables

**Table 1:** List of PCR primers used for qPCR detection of gene expression and bacterial groups.

Gene/bacterial group	Sequences (5′-3′) of primers	Size (bp)	Reference
MUC-2	Forward: GGCTATGGCAGACTTTGT Reverse: GCATTTGCGAGTTATCAG	262 bp	This study
TPH1	Forward: ACCATCTTCCGAGAGCTGAA Reverse: GATGGAAAACCCTGTGCGTT	162 bp	[71]
TPH2	Forward: ATCCCAAGTTTGCTCAGTTTT Reverse: GATGGACGAAAGTAACCCTG	167 bp	[71]
SERT	Forward: AACTGGCAGAAACTCTTGGA Reverse: GAAGATGACGAAGCCAGAGA	195 bp	[71]
*β*-Actin	Forward: TGGCACCACACTTTCTACAAT Reverse: GGTACGACCAGAGGCATACA	189 bp	This study
*Clostridium cluster XIVa*	Forward: AAATGGACGGTACCTGACTAA Reverse: CTTTGAGTTTCATTCTTGCGAA	441 bp	[72]
*Clostridium cluster IV*	Forward: TTACTGGGTGTAAAGGG Reverse: CTTCCTCCGTTTTGTCAA	580 bp	[73]

## Data Availability

The raw data used to support the findings of this study will be made available by the authors, without undue reservation, to any qualified researcher.

## Supplementary Materials

Supplementary Materials Figure S1: composition analysis of gut microbiota at the phylum level (*n* = 4-5). (A) Proportion of *Firmicutes*; (B) proportion of *Bacteroidetes*; (C) the ratio of *Firmicutes* to *Bacteroidetes*. ^∗^*p* < 0.05.
