# Supplementary material for: Administration of a Probiotic Mixture Ameliorates Cisplatin-Induced Mucositis and Pica by Regulating 5-HT in Rats
Source: J Immunol Res. 2021 Sep 14;2021:9321196. doi: 10.1155/2021/9321196 (PMC8461230; doi:10.1155/2021/9321196)
Supplement: Supplementary Materials — Figure S1: composition analysis of gut microbiota at the phylum level (n = 4-5). (A) Proportion of Firmicutes; (B) proportion of Bacteroidetes; (C) the ratio of Firmicutes to Bacteroidetes. ∗p < 0.05. [file 9321196.f1.docx]

Supplemental materials

**Journal name:** Journal of Immunology Research

**Article ID:** 9321196

**Manuscript Title:** Administration of a probiotic mixture ameliorates cisplatin-induced mucositis and pica in rats

**Authors:** Yuanhang Wu, Jianlin Wu, Zhikun Lin, Qian Wang, YingLi, Aman Wang, XiuShan and Jiwei Liu

**Figure S1**

**
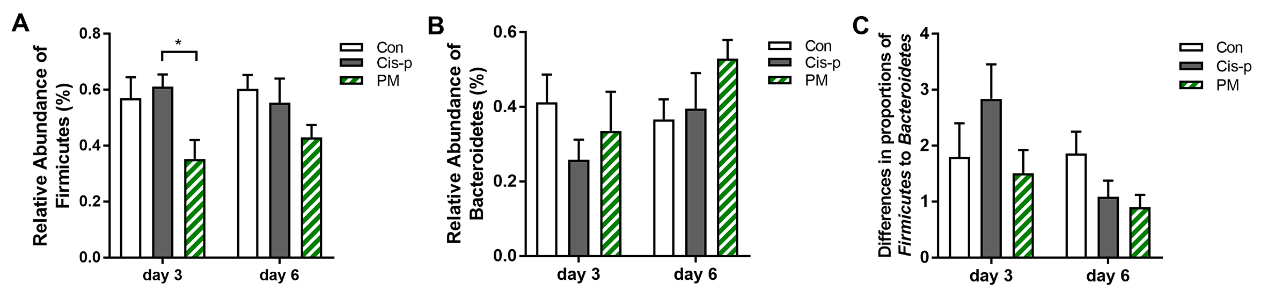
Figure S1**: Composition analysis of gut microbiota at phylum level (n = 4-5). (**A**) Proportion of *Firmicutes*; (**B**) Proportion of *Bacteroidetes*; (**C**) The ratio of *Firmicutes* to *Bacteroidetes*. *p < 0.05.
